# Epidemiology of Bradykinin-mediated angioedema: a systematic investigation of epidemiological studies

**DOI:** 10.1186/s13023-018-0815-5

**Published:** 2018-05-04

**Authors:** Emel Aygören-Pürsün, Markus Magerl, Andreas Maetzel, Marcus Maurer

**Affiliations:** 1 Department for Children and Adolescents, University Hospital Frankfurt, Theodor-Stern-Kai 7, 60596 Frankfurt, Germany; 2 Department of Dermatology and Allergy, Charité – Universitätsmedizin Berlin, Berlin, Germany; 3 Institute of Health Policy, Management & Evaluation, University of Toronto, Toronto, ON Canada; 4 Kalvista Pharmaceuticals, Inc., Cambridge, MA USA

**Keywords:** Angioedema, Bradykinin, Epidemiology, C1-inhibitor, ACE-inhibitor

## Abstract

**Background:**

Bradykinin-mediated angioedema (Bk-AE) can be life-threatening and requires specific targeted therapies. Knowledge of its epidemiology may help optimize its management.

**Methods:**

We systematically searched the medical literature to identify abstracts of interest indexed between 1948 and March, 2016. We used published national survey data on the proportion of the population treated with angiotensin-converting enzyme inhibitors (ACEI) to derive estimates of the population prevalence of ACEI-AE in the USA, Germany and France. For hereditary angioedema (C1-INH-HAE) and C1-inhibitor related acquired angioedema (C1-INH-AAE), publications had to contain original epidemiologic data collection within a defined geographical area. Hereditary angioedema with normal C1-INH was not included in the analysis due to lack of clearly defined criteria.

**Results:**

We identified 4 relevant publications on the prevalence of ACEI-AE, 6 on the prevalence of C1-INH-HAE, and 1 on the prevalence of C1-INH-AAE. The 1st year cumulative incidence of ACEI-AE was estimated to vary between 0.12 (population-based analyses) and 0.30 (meta-analyses of clinical trials) per 100 patient-years. The population prevalence of ACEI-AE was modeled to vary between 7 and 26 in 100,000. The prevalence of C1-INH-HAE was estimated to vary between 1.1 and 1.6 per 100,000. The prevalence of C1-INH-AAE was estimated to be 0.15 per 100,000 in one epidemiological investigation of AAE in Denmark.

**Conclusions:**

Epidemiological evidence on Bk-AE is limited to North America and Europe. ACEI-AE is more common than C1-INH-HAE (~ 10:1), which is more common than C1-INH-AAE (~ 10:1). More studies are needed to comprehensively assess the epidemiological burden of Bk-AE.

## Background

Angioedema is a swelling of the deeper layers of the skin and mucous membranes subsequent to blood vessel dilation and increased vascular permeability induced by vasoactive mediators such as histamine and bradykinin. Angioedema can be life-threatening when involving the upper airways or very debilitating when involving the gastrointestinal tract. Most cases of angioedema are non-hereditary in nature [1]. A significant share is mediated by mast-cell mediators such as histamine, but a substantial proportion are unrelated to mast-cell activation and mediated by bradykinin (Bk-AE). Bradykinin is a potent vasodilator, increases vascular permeability, and mediates pain [2–5]. While mast cell -mediated angioedema can be successfully treated with antihistamines and glucocorticosteroids and with omalizumab as a prophylactic treatment [6], Bk-AE requires interventions that target the synthesis or receptor activity of bradykinin. This distinction is essential in life-threatening situations such as swellings of the upper airways where the use of the wrong therapy translates into unnecessary suffering and the risk of suffocation.

Bk-AE results from a variety of circumstances, such as a slow-down in bradykinin degradation subsequent to the use of angiotensin converting enzyme inhibitor (ACEI-AE), or the uncontrolled generation of bradykinin, such as observed in hereditary angioedema (C1-INH-HAE) and recurrent angioedema due to acquired C1-Inhibitor deficiency (C1-INH-AAE) [7] (Table 1). ACE is critical in the degradation of bradykinin, which is hypothesized to accumulate excessively in some patients taking ACEI for their hypertension. Uncontrolled generation of bradykinin, on the other hand, occurs when complement-1 inhibitor (C1-INH) is either deficient or non-functioning. The physiological role of C1-INH is to control the generation of bradykinin after activation of the contact system. C1-INH-HAE is inherited as an autosomal dominant mutation that results in low levels of C1-INH (type I) or non-functioning C1-INH (type II). An increasing number of patients are also reported to have Bk-AE of hereditary nature, despite normal levels of C1-INH [8, 9]. Mutations of factor XII, and more recently of plasminogen and angiopoietin-1 were found in a small subgroup, but the majority lack clear defining criteria [10, 11]. HAE with normal C1-INH is not covered by our study. C1-INH-AAE is acquired as a result of C1-INH depletion predominantly in association with lymphoproliferative disorders.

**Table 1:** Classification of angioedema (adapted from Craig et al. [7])

Bradykinin-Mediated Angioedema			
C1-INH Deficiency / Defect		C1-INH Normal	
Inherited	Acquired	Inherited	Acquired
HAE-1 & HAE-2	C1-INH-AAE	HAE-3	ACEI-AE

*C1-INH-AAE* acquired angioedema due to C1 inhibitor deficiency, *ACEI-AE* angiotensin-converting enzyme induced angioedema, *HAE-1* hereditary angioedema due to C1 inhibitor deficiency, *HAE-2* hereditary angioedema due to C1 inhibitor defect, *HAE-3* hereditary angioedema with normal C1 inhibitor levels

ACEI-AE is a relatively new phenomenon that is largely determined by the use of ACEI in the population and appears to be twice as likely in patients of African ancestry [12]. The prevalence of C1-INH-HAE should be determined by the incidence of spontaneous mutations, the mortality of the disease, and the average numbers of children of C1-INH-HAE patients. In other words, a stable prevalence would suggest equilibrium among the birth-rates and mortality rates of patients with spontaneously developed and familial C1-INH-HAE.

Prevalence and incidence rates of ACEI-AE and C1-INH-HAE are frequently found in reviews that cover these conditions. For example, the typical prevalence for C1-INH-HAE reported is 1 in 50,000 [13, 14], although the evidence of this has not been systematically assessed. In the present study we report the results of a systematic review of the epidemiological literature on BK-AE and make an attempt of providing evidence-based estimates of its expected prevalence. These estimates are critical in raising awareness among physicians about the differential diagnosis and expected frequency of bradykinin-mediated angioedema [15] and they may help to promote the use of the available targeted treatment approaches that are required for correct medical management of these patients.

## Methods

Two separate search strategies were deployed to obtain publications on the epidemiology of (1) ACEI-AE and (2) C1-INH-HAE and C1-INH-AAE. The following databases were searched:

- Medline from 1948 to March (week 2) 2016
- EMBASE from 1980 to March (week 2) 2016
- The database maintained by the UK NHS Centre for Reviews and Dissemination (CRD) (http://www.crd.york.ac.uk/crdweb/).

The search strategy for ACEI-AE in Medline and EMBASE was the following: exp. angioedema/ AND (angiotensin:.tw. OR ace.tw.) AND (prevalence.tw. OR incidence.tw. OR epidemiol:.tw.), with subsequent deduplication. The search strategy for C1-INH-HAE and C1-INH-AAE was as follows: (*Angio(o)edema/cl, ep, pc OR (hereditary adj angio(o)edema).tw. OR *angio(o)edema, hereditary/ OR (quincke adj angio(o)edema).tw. OR ((acquired adj angio(o)edema).tw.) AND (prevalen: or inciden: or epidemiol:).tw. with subsequent deduplication of references from both databases. The CRD database was searched using the text word combination “angioedema” and “angiotensin” for induced angioedema, and “hereditary angioedema” for C1-INH-HAE.

The titles and abstracts of all references retrieved through the searches were independently scanned by all four authors and had to meet the following criteria to be included: (1) For ACEI-AE the publication had to be a systematic review of randomized controlled trials of ACEI or an inception cohort of new ACEI users; (2) for C1-INH-HAE or C1-INH-AAE the publication had to contain an original epidemiologic data collection in patients with C1-INH-HAE (Type I or II) or C1-INH-AAE, using a survey or sampling strategy that allowed a comprehensive accounting of affected individuals within a defined national area. Discrepancies in the assessments of evaluators were resolved by consensus measures.

To determine the prevalence of ACEI-AE at the population level, a subsequent literature search of Medline and EMBASE was conducted to identify National population-based surveys from, the US, France and Germany that provided information about the proportion of the population being treated with anti-hypertensives and among those, the proportion of patients being prescribed ACEI. Countries representative of large populations and different proportions of ACEi use among antihypertensives were exemplarily selected. The incidence of ACEI-AE from systematic reviews of randomized controlled trials or inception cohort-based epidemiological was then combined with population-based estimates of the use of anti-hypertensives to derive population-based estimates of the incidence or annual prevalence of ACEI-AE. This research is exempt from IRB review.

## Results

### Estimation of the population-based prevalence of ACEI-AE

The search strategy for ACEI-AE in Medline and EMBASE yielded the following results (Table 2):

**Table 2:** Search strategy for ACEI-AE in Medline and EMBASE (OVID) – 1948 to March, week 2, 2016

1	exp angioedema/	20,520
2	angiotensin:.tw	200,463
3	ace.tw	68,287
4	2 or 3	227,296
5	1 and 4	2450
6	(prevalence or incidence or epidemiol:).tw.	2,590,164
7	5 and 6	396
8	limit 7 to review	153
9	7 not 8	243
10	limit 9 to animal	5
11	9 not 10	238
12	after removing duplicates	195

Four relevant publications were retained from the 195 ACEI-AE-related references retrieved in Medline and EMBASE: one study was a pooled analysis of 12 RCTs that compared an ARB with different ACEIs from the manufacturer’s database [16], one was a meta-analysis of 26 US and international randomized controlled trials of ACEI in patients with cardiovascular conditions [12, 16], one was an analysis of new ACEI users in a Medicaid population [17] and one was an original, population-based epidemiological investigation of a tertiary database in the USA [18].

The search for ACEI-AE in the CRD database led to 10 hits: meta-analyses of ACEI-AE (*n* = 2), meta-analysis of ACEI for heart failure (*n* = 1), meta-analyses for ACEI use in primary hypertension (n = 2), tolerability of angiotensin receptor blockers (ARBs) (n = 1), ACEI treatment alone vs. combination therapy with ARBs (n = 1), ARB use in patients with ACEI-AE (n = 1) and unrelated economic evaluations (n = 2). One of the 2 meta-analyses of ACEI-AE found in the CRD database was a protocol for a Cochrane systematic review written in 2008. Only one of the CRD database hits was a relevant meta-analysis, and therefore relevant for our analyses, and this publication was also found in the Medline/EMBASE search [12].

#### Individual study findings (Table 3)

The first meta-analysis was technically a pooled analysis of 12 RCTs comparing telmisartan (*n* = 2564 patients) with ACEI (*n* = 2144 patients) [16]. Angioedema were observed in 4 patients receiving ACEI (0.2%) and no patient receiving telmisartan. The second meta-analysis consisted of a systematic review of Pubmed/CENTRAL and EMBASE from 1980 through Oct. 2011 to find RCTs in patients on ACEI, ARBs or direct renin inhibitors (DRIs) [12]. The authors found 26 trials with 74,857 patients on ACEI (232,523 person-years), 19 trials with 35,479 patients on ARB (122,293 person-years of follow-up), and 2 trials with 5141 patients on DRI (1735 person-years of follow-up). The weighted incidence of angioedema with ACE inhibitors was 0.30% (95% CI 0.28% to 0.32%) compared to 0.11% (95% CI 0.09 to 0.13) with ARBs, 0.13% (95% CI 0.08% to 0.19%) with DRIs, and 0.07% with placebo (95% CI 0.05 to 0.09).

**Table 3:** Tabular summary of retained studies providing estimates of the cumulative incidence of ACEI-AE

Reference	Mancia & Schumacher	Makani et al.	Burkhart et al.	Toh et al.
Region	Global	Global	USA	USA
Time period	1994–2007	1980–2011	1986–1992	2001–2010
Design	Pooled analysis of 12 randomized controlled trials of ACEI vs. ARB from Boehringer Ingelheim database	Syst. review of 26 randomized trials indexed in PubMed, CENTRAL or EMBASE, comparing ACEI vs. placebo or other antihypertensives	Retrospective inception cohort study, Michigan, Ohio and Tennessee Medicaid records, age 15+	Retrospective inception cohort study, age 18+, 17 US health plans in Mini-Sentinel program
Reference population	4708 patients with hypertension, of which 2144 on ACEI	74,857 patients on ACEI, 232,532 patient-years of follow-up	155,258 patients on ACEI, 155,437 patient-years of exposure	65,006,161 health plan members >18y, of which 1,845,138 ACEI initiators
Angioedema Cases	4 on ACEI vs. 0 on ARB	394 on ACEI	285 on ACEI	3301 on ACEI
Diagn. criteria	MedDRA v. 8.1 Adv. event coding	As reported in original trials	ICD 995.1	ICD 995.1
Calculated prevalence	0.2% or 2 per 1000 person-years	0.3% or 3 per 1000 person-years (95% CI: 0.28% to 0.32%)	1.2 per 1000 person-years (adjusted) 1.8 per 1000 (unadjusted)	1.8 per 1000 person-years (0.18%)

The tertiary database analysis provided estimates of the 1st year incidence of ACEI-AE in the US Medicaid population from Michigan, Ohio and Tennessee between 1986 and 1992 [17]. The cumulative incidence in Caucasians varied between 0.9 and 1.3 per 1000 patient-years in the three states (0.09 to 0.13%), while the incidence in blacks varied between 2.8 and 4.2 per 1000 patient-years (0.28 to 0.42%). The pooled incidence across whites and blacks derived from the reported data was 1.2 per 1000 patient-years (adjusted) and 1.8 per 1000 patient-years (unadjusted).

The fourth study provided an estimate of the population-based incidence of ACEI-AE [18] . This was an analysis of the Mini-Sentinel Program instituted by the FDA to develop a national system for monitoring the safety of medical products (2007 FDA Amendments Act). The authors used a new user (inception cohort) design to identify patients > 18 years of age who were prescribed an ACEI, ARB, DRI or beta-blocker (control group) in a 10-year period between Jan 1, 2001 and Dec 31, 2010. The study covered a population base of 65,006,161 health plan members > 18 years old. Angioedema diagnoses of any severity were retained from 3301 patients among 1,845,138 exposed, with 753,105 person-years of follow up. The 365-day cumulative incidence was 1.79 AE cases per 1000 patient years (0.18%). Compared to patients on beta blockers the propensity score adjusted hazard rate ratio for the risk of ACEI-AE was 3.04 (95% CI: 2.81–3.27). None of the ARBs were associated with increased hazard rate ratios vs. beta blockers. However, the use of aliskiren, a direct renin inhibitor (DRI), was associated with an increased hazard rate ratio for AE of 2.85 (95% CI: 1.34–6.04), but only seven patients on aliskiren developed angioedema.

Thus, the 365-day cumulative prevalence of ACEI-AE was estimated to vary between 0.12% (population-based) and 0.30% (RCT-based).

To extrapolate the ACEI-AE incidences to the US population, we used data collected in the National Health and Nutrition Examination Survey (NHANES) of 2009/10 [19, 20]. We multiplied the population-based (0.12%) and RCT-based (0.30%) 365-day cumulative incidence of ACEI-AE with the proportion of ACEI use (33.3%) [20] among the adult (18y+) US population in 2010 (235,153,929) who were estimated to have hypertension (29.1%) and receive antihypertensive treatment (76.4%) for it. Overall, between 20,891 and 52,228 patients were estimated to suffer from ACEI-AE in the US annually, with a population prevalence to vary between 0.7 and 1.7 per 10,000 for 2010 (Table 4).

**Table 4:** Estimated prevalence of ACEI-AE in the US, German and French population supported by population-based estimates of the use of ACEI

	US (2010/11)		Germany (2011)		France (2006/7)	
Description of estimate	%	N	%	N	%	N
Adult population (18+):		235,153,929[35]		67,085,343 [36]		47,911,356 [37]
High blood pressure (18+):	29.1% [19]	68,429,793	31.6% [38]	21,198,968	31.0% [22]	14,852,520
Treated for HBP:	76.4% [19]	52,280,362	71.8% [38]	15,220,859	50.3% [22]	7,470,818
ACEI-use among HBP treated:	33.3% [20]	17,409,361	45.0% [21]	6,849,387	26.7% [39]	1,994,708
Estimated number of patients with ACEI-AE						
(low – 0.12%):		20,891		8219		2394
(high – 0.30%):		52,228		20,548		5984
Population (all ages):		309,349,689[35]		80,219,695 [36]		61,538,322 [37]
Prev. (# in 10,000) of ACEI-AE in the population:						
low		0.7		1.0		0.4
high		1.7		2.6		1.0

For Germany we found two publications providing data on the prevalence of hypertension in general as well as treated hypertension [20] and the use of ACEI [21]. The annual population-based prevalence of ACEI-AE was derived based on a multiplication of the population-based (0.12%) and RCT-based (0.30%) prevalence of ACEI-AE with the proportion of ACEI use (45.0%) among users of antihypertensive drugs in the adult population of patients with hypertension (31.6%) among which 71.8% were considered treated. The resulting prevalence estimate was calculated to vary between 1.0 and 2.6 per 10,000 general inhabitants for the year 2011. In comparison, Germany had the highest ACEI-AE prevalence. The prevalence of ACEI-AE is determined by the extent of ACEI use in a population. Germany not only has a relatively high proportion of treated hypertension, but also has the highest relative share of ACEI in the treatment of high blood pressure, which both might explain our finding.

For France, the annual population-based prevalence of ACEI-AE was derived based on a multiplication of the population-based (0.12%) and RCT-based (0.30%) prevalence of angioedema in ACEI users with the proportion of ACEI use (26.7%) [22] among users of antihypertensive drugs in the adult population of patients with hypertension (31.0%) of which 50.3% were considered treated [22]. Based on these data, the annual prevalence of ACEI induced AE in France was estimated to vary between 0.4 and 1.0 in 10,000 given the results of the French National Health Nutrition Study (ENNS 2006–2007), the first study using blood pressure readings taken in a national sample of adults aged 18–74 years and living in continental France.

### Estimation of the population-based prevalence of C1-INH-HAE and C1-INH-AAE

The search strategy for C1-INH-HAE and C1-INH-AAE (Table 5) resulted in 407 potentially relevant references for C1-INH-HAE and C1-INH-AAE. The search of the CRD database led to the retrieval of five HTAs:

1. C1-inhibitor Berinert® by the All Wales Therapeutics and Toxicology Centre (AWTTC) in 2013
2. C1-inhibitor Cinryze® by AWTTC in 2013
3. Icatibant Firazyr® by AWTTC in 2012
4. Conestat alfa (Ruconest®) by the UK National Horizon Scanning Centre (NHSC) in 2010 and
5. Icatibant (Firazyr®) by NHSC in 2008.

**Table 5:** Search strategy for C1-INH-HAE and C1-INH-AAE in Medline and EMBASE (OVID)

1	Angioedema/cl, ep, pc [Classification,Epidemiology, Prevention & Control]	346
2	(hereditary adj angioedema).tw. or *angioedema, hereditary/ or (quincke adj angioedema).tw.	7489
3	(hereditary adj angiooedema).tw. or *angiooedema, hereditary/ or (quincke adj angiooedema).tw.	5797
4	(acquired adj angioedema).tw. or (acquired adj angiooedema).tw.	435
5	or/1–4	7694
6	(prevalen: or inciden: or epidemiol:).tw.	2,586,905
7	5 and 6	500
7	remove duplicates from 6	407

No systematic reviews or published economic evaluations were found. The Cochrane Library partially cross-references the databases maintained by CRD. The same two HTAs were identified in the Cochrane collaboration database.

Six prospective, population-based epidemiological investigations of C1-INH-HAE published as full-length publications were found, and one prospective population-based epidemiological investigation published as a conference abstract. Each study is summarized below and in Table 6.

**Table 6:** Population-based epidemiological investigations of C1-INH-HAE

Reference	Roche et al.	Stray-Pedersen et al.	Bygum A	Nordenfelt et al.	Zanicchelli et al.	Psarros et al.
Region	Spain	Norway	Denmark	Sweden	Italy	Greece
Time period	1999–2004	1998/1999	1999, 2003/4 and 2009	2011	1973–2013	2010–2013
Design	Doctor, hospital and patient survey	Hospital & patient survey, telephone follow-up	Comprehensive national survey (hospitals, doctors, laboratories, media and patient incl. families)	Sweha-Reg, a population-based census of HAE in Sweden	Survey of 17 dedicated tertiary referral centers	Prospective HAE registry
Reference population	Spain 2001, 40.476 Millions	Norway 1999, 4.462 Millions	Denmark 2009, 5.523 Millions	Sweden 2010, 9.348 Millions	Italy 2013, 60.783 Millions	Greece, 2012 10.815 Millions
Cases	444	67	76	146	983	116
Diagn. criteria	Criteria & C1-INH	Diagnosis as per record	Criteria & C1-INH	Criteria & C1-INH/C4	Criteria & C1-INH	Criteria & C1-INH
Calculated prevalence	1.10 per 100,000 / 1 in 91,162	1.50 per 100,000 / 1 in 66,597	1.38 per 100,000 / 1 in 72,671	1.56 per 100,000 / 1 in 64,028	1.51 per 100,000 / 1 in 66,284	1.07 per 100,000 / 1 in 93,235

#### Prevalence of C1-INH-HAE in Spain

This study was based on a mail-survey that was sent to allergist and immunologist practitioners in Spain in 1999 and was subsequently followed up by telephone [23]. Hospitals and centers potentially treating patients with C1-INH-HAE were also contacted by mail. Patients were also referred to the investigators by the Spanish association of patients with HAE. All patients recorded in the registry through the various sampling approaches were contacted by telephone between 2003 and 2004. Only patients with Type I or II HAE confirmed by laboratory testing were included in this study. Patients with acquired C1-INH deficiency or angioedema and no functional or antigenic C1-INH deficiency were excluded. Genetic studies were conducted on approximately three quarters of the subjects.

Four hundred and forty-four patients were identified, belonging to 135 unrelated families. Twenty-seven patients had no family history of HAE. The authors calculated a prevalence of C1-INH-HAE of 1.09 per 100,000 inhabitants (1 in 92,000), based on a population of 40.9 million people living in Spain in 2001. The authors considered this a “minimal prevalence” given the strategy of sampling through tertiary referral centers. The proportion of asymptomatic patients who had never had an angioedema episode was 13.7%. The study also identified 19 previously undiagnosed patients and four patients who were falsely diagnosed with C1-INH-HAE, but had other forms of angioedema.

#### Prevalence of C1-INH-HAE in Norway

The prevalence of C1-INH-HAE in Norway was investigated using a research registry of patients with primary immune deficiency [24]. Patients with either a suspected or established diagnosis were entered into the registry. The survey was conducted in 1998/1999 among all non-psychiatric hospitals of Norway (140 departments in 60 hospitals). Questionnaires were also distributed to members of patient and professional organizations. Reminders were sent to non-responders. Telephone follow-up was conducted where necessary. Diagnoses were made according to World Health Organization criteria. Information reported for patients with C1-INH deficiencies included gender and year of birth.

Complement deficiencies were reported by 78 patients, of whom 67 had C1-INH deficiency. Based on a population in Norway of 4.46 million people in 1999, this translates to a prevalence of 1.75 per 100,000 for all complement deficiencies and a prevalence of 1.5 per 100,000 (1 in 67,000) for C1-INH deficiency.

#### Prevalence of C1-INH-HAE in Denmark

A comprehensive national survey of C1-INH-HAE was conducted in Denmark in 2001, and repeated again in 2007–2008 [25]. An extensive recruitment strategy was initiated following the establishment of a national comprehensive care center for patients with HAE. The recruitment strategy targeted hospital departments, dermatologists, centers for rare diseases, patient organizations and Danish national reference laboratories. The survey was advertised through local and national media outlets and complemented with family interviews and examination of medical records.

The diagnosis of C1-INH-HAE was based on functional or antigenic values of C1-INH of less than 50% on two separate instances, on the background of clinical symptoms or a family history of HAE. The authors identified 82 patients with C1-INH-HAE, comprising 77 with HAE Type I and 5 with HAE Type II in 26 families. Eleven patients were without affected family members. The prevalence of C1-INH-HAE was calculated based on a Danish population of 5.4 million inhabitants in 2009. However, by this time two of the original 82 HAE patients had died and four others had emigrated for a final total of 76 patients. Based on this number, the prevalence of C1-INH-HAE in Denmark was estimated to be approximately 1.41 cases per 100,000 (1 in ~ 71,000).

The authors were confident that the calculated prevalence is likely to be the true prevalence of patients with C1-INH-HAE in Denmark, given the comprehensive sampling strategy, the consultation of the national reference laboratory, and the screening of family members. However, they acknowledge that patients diagnosed before 1993 without any further follow-up could have been missed by the survey.

#### Prevalence in Sweden

A retrospective patient survey was conducted in Sweden to investigate the disease burden of C1-INH-HAE using Sweha-Reg, a population-based census of C1-INH-HAE patients [26]. All known C1-INH-HAE patients in Sweden were invited to participate in the study. Patients were contacted by the Swedish Patient Organization or by one of the two special laboratories for complement deficiency, or departments of internal medicine, otorhinolaryngology, allergy, dermatology, or pediatrics known to treat patients with HAE. One hundred and forty-five patients were identified within the database as of June 2011, leading to a calculated minimal prevalence in Sweden of 1.51 per 100,000 (1 in 66,000), given a population size of 9.348 Million.

#### Prevalence in Italy

Patients registered by the Italian network for C1-INH-HAE (ITACA) from 17 tertiary referral practices were included in the study [27]. Diagnosis of HAE Type 1 and 2 was made on the basis of the patient’s personal or family history and on C1-INH functional or antigenic plasma levels ≤50% of normal. Nine-hundred eighty three (983) patients were identified, of which 63 had died and 3 were missing critical information. Therefore, the prevalence of HAE Type 1 and 2 in Italy was estimated to be at least 1.51 per 100,000 (1 in 66,284) at the end of 2013, when the Italian population consisted of 60,782,668 inhabitants.

#### Prevalence in Greece

A national registry of patients with HAE was initiated in Greece in 2009 [28]. This was an extensive effort of recruitment adopted by the Hellenic Society for Allergy and Clinical Immunology. Patients recruited into the registry between 7/2010 and 6/2013 were included in the publication. Confirmatory laboratory tests were conducted to establish the diagnosis on the basis of the International Consensus publication. One-hundred sixteen (116) patients were identified, establishing a prevalence of 1.07 per 100,000, or 1 in 93,235 for the 2012 population of Greece, which then had 10,815,197 inhabitants.

### Prevalence of C1-INH-AAE

Only one study provided an original estimation of the population-based prevalence of C1-INH-AAE. This study was undertaken in Denmark and identified eight (8) patients with C1-INH-AAE in the entire nation. According to the authors, this number represents less than 10% of the C1-INH-HAE patients, as there were 82 patients with C1-INH-HAE identified in Denmark, a percentage that is in line with a previous observation of a 1:10 ratio of C1-INH-AAE: C1-INH-HAE [29].

## Discussion

We summarized the studies reporting on the annual prevalence of ACEI-AE, and prevalence of C1-INH-HAE and C1-INH-AAE. The prevalence of ACEI-AE is determined by the country-specific use of antihypertensive; in the US, the prevalence was estimated to vary between 0.7 and 1.7 per 10,000 inhabitants when weighting the risk of ACEI-AE among Caucasians and African-Americans. In Germany it was estimated to vary between 1.0 and 2.6 per 10,000 while in France it was estimated to be lower and vary between 0.4 and 1.0 in 10,000. The prevalence of C1-INH-HAE was estimated to vary between 1.1 and 1.6 per 100,000. The prevalence of C1-INH-AAE was estimated to be 1.5 per 1 Million from the one available epidemiological investigation of C1-INH-AAE in Denmark.

Overall, the prevalence figures would be approximately 1.5 per 10,000 for ACEI-AEs, 1.5 per 100,000 for C1-INH-HAE and 1.5 per 1 Million for C1-INH-AAE. For every 100 patients with ACEI-AE there would be 10 patients with C1-INH-HAE and 1 patient with C1-INH-AAE. However, patients with C1-INH-HAE have on average 1 angioedema attack per month [25]. For this reason, the number of annual angioedema attacks due to C1-INH-HAE and ACEI use should be similar, given that ACEI are typically withdrawn at the time of diagnosis and re-occurrence is less likely. For a country with 100 Million inhabitants there would be approximately 31,500 bradykinin mediated angioedema attacks each year, calculated by multiplying the prevalence standardized to 1 million, 150 for ACEI-AE, 15 per for C1-INH-HAE, and 1.5 for C1-INH-AAE, with 100 for each entity, and multiplying the resulting figures with an annual attack rate of approximately 1 per year for ACEI-AE and 10/year for C1-INH-HAE and C1-INH-AAE.

Treatments for histamine-mediated angioedema are inappropriate for bradykinin-mediated angioedema. These numbers highlight the significant need to recognize the nature of the underlying cause in each patient who represents to the emergency room with signs and symptoms of an angioedema attack.

In the absence of confirmatory investigations, many publications referenced the prevalence of C1-INH-HAE to be 1 in 10,000 to 1 in 50,000. Several studies have now reported the population-based prevalence of C1-INH-HAE. These studies were supported by intense efforts of patient societies and providers to register patients in order to provide access to adequate treatment and also to raise awareness and shorten the delay to a correct diagnosis. We have summarized these studies and determined that the prevalence of diagnosed cases of C1-INH-HAE is approximately 1.5 per 100,000 (or ~ 1 in 67,000).

A population-based estimate of ACEI-AEs has not been published before. The annual (1st-year) cumulative incidence of angioedema among ACEI users is fairly stable, as the meta-analyses of the randomized controlled trials and the database analyses suggest. However, the population-based prevalence is necessarily variable and depends on the actual use of ACEI in the population, how this use was measured, and the demographic mix, especially with respect to the representation of African-Americans. For this reason we were only able to provide approximate estimates with potentially large variation. Other antihypertensives addressing the renin-angiotensin system such as angiotensin-receptor blockers or direct renin inhibitors have not been conclusively linked to increased risk of AE. Any incremental risk of AE in users of such drugs would translate into a higher prevalence in the population. Also, because the ratio of patients with ACEI-AE vs. C1-INH-HAE is approximately 10:1, we calculated that the expected number of angioedema attacks is similar, due to the frequency of attacks among C1-INH-HAE patients.

We searched both the Medline and EMBASE database using appropriate keywords to select epidemiological investigations and systematic reviews. A cross-check of the CRD database confirmed that no systematic reviews of ACEI were missed. The C1-INH-HAE-associated literature is not as extensive as can be seen from the overall number of search hits, because the disease is rare and is covered by a small community of researchers. Because there were not so many publications, we did not attempt to quantitatively combine the estimates, but rather describe each individual study. This will enable the readers to come to their own conclusion regarding the manifested prevalence of C1-INH-HAE or ACEI-AE in their own setting.

It appears that the epidemiology of C1-INH-HAE is stable across the geographic regions covered in this review. This may be the result of a stable mix of spontaneous mutations and inherited patterns [30, 31]. It is likely that the prevalence of 1 in 67,000 provides a reference ceiling to the estimation of diagnosed patients with C1-INH-HAE in a specific country, given the current pattern of detection in countries with modern healthcare systems. This number might, therefore, be helpful to policymakers in estimating the number of patients expected in their constituencies. Moreover, as investigations of family members of affected patients have shown [32], there still persists a percentage of undiagnosed family members. Further efforts in raising disease awareness, educating providers and improving access to appropriate therapies may shorten the current lagtime of 10 [33] to 16 [25] years between onset of first symptoms and date of diagnosis.

There are several potential clinical implications of our results: ACEI-AE may currently be underdiagnosed in some populations. Up until 2017, the German national database on suspected adverse drug reactions listed about 2000 angioedema incidents in connection with ACEI use while prescription rates of ACEI-AE suggest a considerably larger number [34]. This points to a gap in the diagnosis of angioedema among ACEI users and highlights the need for expanding the knowledge on clinical findings, course and potential therapy of acute ACEI-AE in the medical community. Vice versa, there are factors that promote potential misdiagnosis of C1INH deficiency cases as ACEI-AE cases: ACEI use is quite common in many populations, At the same time, ACEI are a common precipitating factor of acute angioedema in patients with C1-INH deficiency. In undiagnosed C1-INH deficiency cases, an angioedema episode in the presence of ACEI therapy would most likely be attributed to the ACEI. This might lead to a, probably minor, effect on prevalence numbers of C1-INH-HAE and C1-INH-AAE. The recognition of an ACEI-AE should prompt the exclusion of C1-INH deficiency as in diagnosed C1-INH deficiency cases investigation of family members is warranted. To characterize clinical differences in these different entities more deeply is an unmet need and will be the goal of future efforts.

Limitations of the study include its inherent restriction to US and European data, while the epidemiology of Bk-AE might be different in other parts of the world. Also, the prevalence of ACEI-AE may be higher as estimated in our analysis as in some of the studies patients with previous angioedema were excluded [12, 18]. A few trials did not report incidence of angioedema, as adverse effects were only reported if the incidence was > 1% [12]. Another limitation is the low number of prevalence studies in Europe, for example but not restricted to Germany and France that a lack systematic nationwide data collection. Additionally, the present analysis is focused on ACEI-AE and C1-INH-HAE. Further efforts to assess the epidemiology of additional types of HAE with normal C1-INH, for example HAE due to a FXII mutation (FXII-HAE), are needed. It has yet to be determined if angioedema attacks in patients with HAE due to a novel plasminogen mutation (HAE-PLG), HAE with a novel angiopoietin-1 gene mutation (ANGPT-1-HAE) or HAE of unknown origin (U-HAE) are bradykinin mediated, in which case future analysis of Bk-AE epidemiology should include these novel types. A further limitation of the present analysis might be the fact that the studies indentified essentially covered the last two decades. As awareness of Bk-AE increased considerably over time, there is a chance that older prevalence data may underestimate the real prevalence. Continued efforts in assessing the prevalence of Bk-AE are therefore warranted.

## Conclusion

Epidemiological evidence on Bk-AE is largely limited to North America and Europe. ACEI-AE is more common than C1-INH-HAE (10:1), which is more common than C1-INH-AAE (10:1). More studies are needed to comprehensively assess the epidemiological burden of Bk-AE.

## Abbreviations

ACE: Angiotensin converting enzyme
ACEI: Angiotensin-converting enzyme inhibitor
ACEI-AE: Angiotensin-converting enzyme inhibitor related angioedema
ARB: Angiotensin receptor blocker
AWTTC: All Wales Therapeutics and Toxicology Centre
Bk-AE: Bradykinin mediated angioedema
C1-INH: C1 inhibitor
C1-INH-AAE: acquired angioedema with C1 inhibitor deficiency
C1-INH-HAE: hereditary angioedema with C1 inhibitor deficiency
CI: Confidence interval
CRD: Centre for Reviews and Dissemination, University of York
DRI: Direct renin inhibitor
FDA: US Food and Drug Administration
HAE: Hereditary angioedema
HBP: High blood pressure
HTA: Health technology assessment
ICD: International Statistical Classification of Diseases
NHANES: National Health and Nutrition Examination Survey
NHSC: National Horizon Scanning Centre
RCT: Randomized controlled trial
